# The B.M.A. Annual Representative Meeting

**Published:** 1917-07-28

**Authors:** 


### The Hospital

The Workers' Newspaper of Administrative Medicine and Institutiona Life, Administration, National Insurance and Health.

No. 1625, Vol. LXII. SATURDAY, JULY 28, 1917. PRICE ONE PENNY

### THE B.M.A. ANNUAL REPRESENTATIVE MEETING.

As in 1915, and again in 1916, the annual meeting of the British Medical Association will be unable to boast either the scientific or the social attractions which have in earlier years formed important parts of its programme. The times forbid such enterprises, and prudence and good taste allow the claim. But the imperious demands of business may not be stayed, and hence on this side the activities of the Association continue. These take the form of what it is customary to call medical politics, and to deal with them is the duty of that part of the organisation known as the Representative Body. This consists of "representatives" appointed by the Divisions of the Association through the length and breadth of the land, and the true position of these "representatives" is that of delegates. In other words, they are sent by their several constituencies, not to follow an independent judgment, but to vote in accordance with definite instructions on the various questions which are to engage the attention of the Representative Body. One further word on official machinery may be added. It is that a vote of the Representative Body is the legal voice of the Association and is binding on the members of the Association. The Council itself is governed by such votes, and its executive actions must be in accordance with them. Thus the meeting of the Representative Body is of supreme interest to the Association, for its decisions determine and command policy. They stand firm and may not be challenged.

It may be fairly asked whether the proceedings of the Representative Body can claim to be the voice not merely of the Association but also of the profession, and the question is not one which can be answered with confidence. It is true that the delegates who assemble in the Representative meeting speak nominally for the various parts of the country and that their position has thus an appearance of authority. But there are certain qualifications of this position. In the first place the Association does not include the whole profession. Many practitioners remain outside it, and are either hostile to its policy or indifferent to it. In some instances there is a blunt refusal to have anything to do with medical politics; in others the Association is regarded as of little value. Again, it is notorious that the meetings of the Divisions at which the delegates are appointed and instructed are but poorly attended, and this necessarily diminishes the representative character which such delegates appear to assume. A pleasant self-satisfaction may argue that the abstention of members from the Divisional meetings means that they are quite content to leave affairs in the hands of the interested few. But another interpretation is obviously possible, and the candid friend may equally say that such abstention means a conviction that the proceedings of the Association have no practical fruition. Further, it must be allowed that with few exceptions the members of the Representative Body are not drawn from practitioners who can legitimately boast a position of authority or leadership in the profession. For some reason or other men of this order, at least speaking generally, do not take any conspicuous interest in the medico-political work of the Association. With its scientific activities they may be in full sympathy, but in its relation to legislative and social measures they show little or no concern. Take these various considerations into full view and there is a serious diminution to the claim of the Representative Body to voice and express the opinion of the profession. It has its value, but is not sufficiently broad-based to be conclusive. But there is still more to be said. Even among the active adherents of the Association anything but unity and good will prevails. The bitter quarrel of panel and non-panel practitioners may have ceased its more active manifestations, but a dull hostility still continues, and on occasions its murmur takes a louder tone. It exists, and with it a reckoning has to be held. Without any doubt at all this quarrel is a serious matter for the Association, for a schism either of one party or the other would mean most unpleasant consequences. We hold no brief for the Association, and upon occasions not a few we have dissented most strongly from its policy. But we have a very active regard for the interests and welfare of the medical profession, and we are anxious that it should have a legitimate organ to speak not only for its own interests, but also on behalf of the still wider interests committed to its care. It is a loss to the nation when the informed view of the medical profession has not a just opportunity to express itself upon all questions which touch the national health, and there is an added regret when such an opportunity is closed by the action of the profession itself. Hence we should hear with pleasure that all internal and domestic strife had ceased, and that from all orders and ranks of the profession a contribution could be secured towards the solution of the problems which exist to-day or which will present themselves in the immediate future. If this is to be obtained, a fair spirit of discussion and of reasonable compromise must be promoted. The more fortunate and distinguished men must pay some heed to the needs and trials of their less happily situated colleagues, and must not dwell in lofty isolation separate and detached from cares which do not touch them personally. Only by union and by the cultivation of community of interest can the medical profession take its due place in the national counsels, and, for our own part, we are little concerned with the means of expression so long only as this has a truly representative and authoritative voice.

